# Design and Optimization of the Resonator in a Resonant Accelerometer Based on Mode and Frequency Analysis

**DOI:** 10.3390/mi12050530

**Published:** 2021-05-07

**Authors:** Yan Li, Biao Jin, Mengyu Zhao, Fuling Yang

**Affiliations:** School of Mechanical Electronic & Information Engineering, China University of Mining and Technology-Beijing, Beijing 100083, China; 201572@cumtb.edu.cn (Y.L.); jinbiao@student.cumtb.edu.cn (B.J.); zmy@student.cumtb.edu.cn (M.Z.)

**Keywords:** resonant accelerometer, resonator, mode, frequency, design and optimization

## Abstract

This study aims to develop methods to design and optimize the resonator in a resonant accelerometer based on mode and frequency analysis. First, according to the working principle of a resonant accelerometer, the resonator is divided into three parts: beam I, beam II, and beam III. Using Hamilton’s principle, the undamped dynamic control equation and the ordinary differential dynamic equation of the resonant beam are obtained. Moreover, the structural parameters of the accelerometer are designed and optimized by using resonator mode and frequency analysis, then using finite element simulation to verify it. Finally, 1 g acceleration tumbling experiments are built to verify the feasibility of the proposed design and optimization method. The experimental results demonstrate that the proposed accelerometer has a sensitivity of 98 Hz/g, a resolution of 0.917 mg, and a bias stability of 1.323 mg/h. The research findings suggest that according to the resonator mode and frequency analysis, the values of the resonator structural parameters are determined so that the working mode of the resonator is far away from the interference mode and avoids resonance points effectively. The research results are expected to be beneficial for a practical resonant sensor design.

## 1. Introduction

A resonant accelerometer converts acceleration directly into a frequency, it benefits from the advantages of high measurement accuracy, strong anti-disturbance capability, good stability, wide dynamic range, and quasi-digital output [1,2,3,4,5]. A resonator is a device designed to vibrate at a specified frequency. The specific frequency is one where the system retains input energy with minimum loss, which is one of its natural frequencies [6,7]. A resonator is the basis for combining many devices, such as radio frequency filters and resonant sensors [8,9,10]. In a resonant accelerometer, the resonator is the core sensitive element [11,12]. Thus, the mode and frequency of the resonator affect the performance of the resonant accelerometer greatly [13,14].

In their study on resonator mode, Yang and Huang proposed an analytical model to obtain a higher order mode in a rectangular resonator [15]. Phani et al. studied the modal coupling of the z-axis frame vibration gyro [16]. Guo et al. calculated the modal frequency and quality factor for the square resonant cavity [17]. The effect of dispersive modal coupling to tune the frequency difference between two coupled modes in a wide range was studied by Lu et al. [18]. In terms of resonator frequency, Hassanpour et al. studied the influence of axial force, position, mass ratio, and radius of rotation on the natural frequency and resonator mode [19]. A new method was proposed to improve the resonator’s frequency stability by Du et al. [20]. Huang et al. found that the displacement due to warpage brings the natural frequency closer to the real natural frequency of the resonator [21]. Guzman et al. developed a SiC bridge resonator with an unprecedentedly large tunable frequency range [22]. The aforementioned studies mainly investigated the frequency error of the coupled mode and its effect on the resonator. However, none of the studies examined how the mode influences the structural parameters and sensitivity of the resonator explicitly.

To design the structure parameters of the resonator, Shi et al. studied the gap sizes and the excitation parameters and their effect on the resonator performance by using the uncertainty method [23]. Zotov et al. proposed a dynamic equilibrium method to maximize the quality factor in the defect structure of the resonator [24]. Candler et al. demonstrated the effect of the curved resonant beam gap on the quality factor [25]. Zhou et al. used a multi-objective optimization method to design the resonator structure parameters [26]. As for the resonator sensitivity, the influence of the structure parameters on resonator sensitivity was studied by Shi et al. [27]. Xu et al. designed a new structure with a suspended piezoresistive beam (SPB) to improve sensitivity without sacrificing the resonant frequency [28]. Du et al. used the twinborn diaphragms to improve the resonator sensitivity [29]. Zhao et al. used three weakly coupled resonators to improve sensitivity to stiffness changes [30]. The aforementioned studies mainly focused on the influence of the structure parameters on the resonator sensitivity but did not propose a new structure parameter optimization method to improve the sensitivity.

Equating the resonator to a single resonant beam is an effective way to study the dynamic characteristics of the resonator in resonant sensors. Our previous work has established a dynamic resonant beam model and analyzed its dynamic characteristics [31]. In addition, the stability analysis method and vibration excitation parameter characteristics have been proposed based on a single resonant beam model [32,33].

This simplified model that equates the resonator to a single resonant beam is convenient for analysis, and its force–frequency characteristics can be found easily. Unfortunately, there is an error with the real working conditions. In the present study, the displacement model of the resonant beam is established to reduce this error, and the Hamilton principle is used to establish a partial differential dynamic control equation. The linear mode and frequency are solved, which is the quantitative analysis being used to design the resonator, and a series of experiments being carried out to prove the feasibility of the theoretical analysis method.

## 2. Theory of the Resonator

### 2.1. Working Mechanism

**Figure 1:**
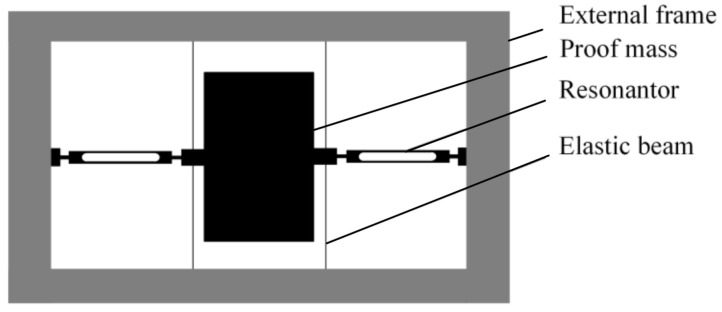
Schematic of the resonant accelerometer.

The resonant accelerometer structure is shown in Figure 1, consisting mainly of an external frame, a proof mass, two resonators, and four elastic beams. Each of the resonators is actuated at a resonance using drives in this particular implementation. The external acceleration applied to the proof mass along the sensitive axis results in an inertial force transmitted axially to the resonator. The applied axial force results in a shift in the resonant frequency of the resonator. This effect is the same as tuning a guitar string to resonate at different frequencies by varying the tension in the string. The output of the accelerometer is the difference between the output frequency of the two resonators.

**Figure 2:**
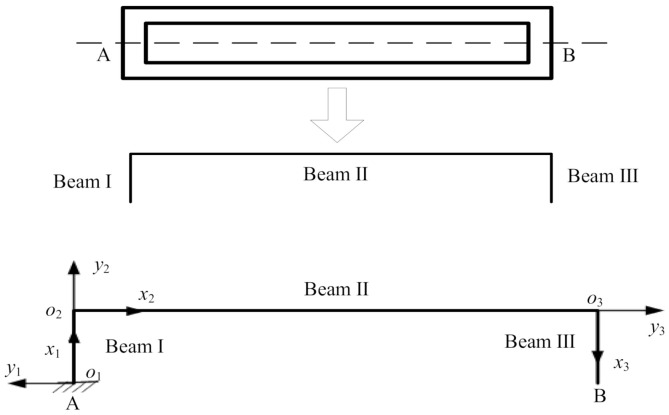
Simplified resonator implementation.

In the resonator working mode, the local vibration mode of its upper and lower beam is the shape of the reverse half wave. As shown in Figure 2, the smaller the displacement of the intersection of the structure and the dotted line, the higher the energy of the excitation that is transferred to the working mode. Thus, in order to facilitate the analysis in the working mode, the dynamic characteristics of half of the resonator structure can be analyzed based on the assumption that the intersection points A and B are static.

The resonator model is simplified to be made up of beam I, beam II, and beam III, as shown in Figure 2. As a function of the applied acceleration, the response of the resonant beam to the external axial force is to bend and twist. The main response of its lower order vibration is to bend. The effects of the changing external force result in a bending vibration in the plane of the three beams. In the simplified resonator model, one end of each resonant beam is fixed and the other end is free; beams I and III are kept parallel; and the length, width, and thickness of the beams are variable parameters that can be used to describe different resonator states.

### 2.2. Vibrational Mode and Frequency

**Figure 3:**
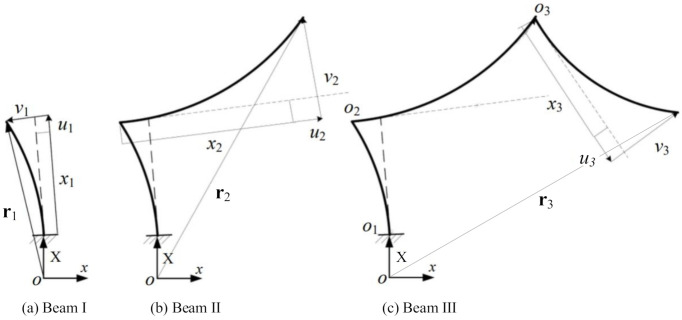
Schematic of the displacement of the resonant beam.

Beam III is parallel to beam I prior to the deformation of the resonator structure. As shown in Figure 3, $o_{1}x_{1}y_{1}$ is the local coordinate of beam I, which is consolidated at the left end of beam I. $o_{2}x_{2}y_{2}$ is the local coordinate of beam II, which is consolidated at the left end of beam II. $o_{3}x_{3}y_{3}$ is the local coordinate of beam III, which is consolidated at the left end of beam III. In the local coordinate $o_{i}x_{i}y_{i}$, the lateral displacement of the beam is $v_{i}$ and the axial displacement is $u_{i}$. In the global coordinate oxy, the displacement of the resonant beam is $r_{i}$.

The kinetic energy expression of each section of the resonator is as follows:

(1) $$T_{i}=\int_{0}^{L_{i}}\int_{A_{i}}\frac{1}{2}\rho_{i}{\left|{\dot{r}}_{i}\right|}^{2}dA_{i}dx_{i}=\int_{0}^{L_{i}}h_{T_{i}}dx_{i},i\in \left\{1,2,3\right\},$$

where the dots indicate derivation with time *t*, $L_{i}$ is the length of each beam, $\rho_{i}$ is the density of each beam, $A_{i}$ is the cross-sectional area of each beam.

The potential energy expression of each section of the resonator is as follows:

(2) $$V_{i}=\int_{0}^{L_{i}}\int_{A_{i}}\frac{\varepsilon_{i}\sigma_{i}}{2}dA_{i}dx_{i}=\int_{0}^{L_{i}}h_{V_{i}}dx_{i},i\in \left\{1,2,3\right\},$$

where $\varepsilon_{i}$ is the axial line strain of each beam, $\sigma_{i}$ is the axial positive stress of each beam. The expressions are as follows:

(3) $$\varepsilon_{i}={u^{\prime }}_{i}-y_{i}{v^{\prime \prime }}_{i},\sigma_{i}=E\varepsilon_{i},$$

Using the Hamilton principle,

(4) $$\int_{t_{1}}^{t_{2}}\sum_{i=1}^{3}\left(\delta T_{i}-\delta V_{i}\right)dt=0,$$

The dynamic control equation can be expressed as follows by the energy density function:

(5) $$\frac{\partial^{2}h_{T_{i}}}{\partial {\dot{u}}_{i}\partial t}-\frac{\partial^{2}h_{V_{i}}}{\partial {u^{\prime }}_{i}\partial x_{i}}=0,\frac{\partial^{2}h_{T_{i}}}{\partial {\dot{v}}_{i}\partial t}+\frac{\partial^{3}h_{V_{i}}}{\partial {v^{\prime \prime }}_{i}\partial x_{i}^{2}}=0,i\in \left\{1,2,3\right\},$$

In the equation, the six unknown functions represent the lateral and axial displacements of the three resonant beams in the resonator. Expressions of the 18 boundary conditions corresponding to the motion control in Equation (5) are described as follows:

(6) $$\left[\sum_{i=1}^{3}\int_{0}^{L_{i}}{\frac{\partial^{2}h_{T_{i}}}{\partial {\dot{u}}_{1}\left(L_{1}\right)\partial t}dx_{i}-\frac{\partial h_{V_{1}}}{\partial {u^{\prime }}_{1}}|}_{x_{1}=L_{1}}\right]\delta u_{1}\left(L_{1}\right)=0,{\frac{\partial h_{V_{i}}}{\partial {u^{\prime }}_{i}}\delta u_{i}|}_{x_{i}=0}=0,$$

(7) $$\left[{\frac{\partial h_{V_{2}}}{\partial {u^{\prime }}_{2}}|}_{x_{2}=L_{2}}+\int_{0}^{L_{3}}\frac{\partial^{2}h_{T_{3}}}{\partial {\dot{u}}_{2}\left(L_{2}\right)\partial t}dx_{3}\right]\delta u_{2}\left(L_{2}\right)=0,{\frac{\partial h_{V_{3}}}{\partial {u^{\prime }}_{3}}\delta u_{3}|}_{x_{3}=L_{3}}=0,$$

(8) $$\left[{\frac{\partial^{2}h_{V_{1}}}{\partial {v^{\prime \prime }}_{1}\partial x_{1}}|}_{x_{1}=L_{1}}-\sum_{i=1}^{3}\int_{0}^{L_{i}}\frac{\partial^{2}h_{T_{i}}}{\partial {\dot{v}}_{1}\left(L_{1}\right)\partial t}dx_{i}\right]\delta v_{1}\left(L_{1}\right)=0,{\frac{\partial^{2}h_{V_{i}}}{\partial {v^{\prime \prime }}_{i}\partial x_{i}}\delta v_{i}|}_{x_{i}=0}=0,$$

(9) $$\left[\sum_{i=1}^{3}\int_{0}^{L_{i}}{\frac{\partial^{2}h_{T_{i}}}{\partial {{\dot{v}}^{\prime }}_{1}\left(L_{1}\right)\partial t}dx_{i}+\frac{\partial h_{V_{1}}}{\partial {v^{\prime \prime }}_{1}}\delta {v^{\prime }}_{1}|}_{x_{1}=L_{1}}\right]\delta {v^{\prime }}_{1}\left(L_{1}\right)=0,{\frac{\partial h_{V_{i}}}{\partial {v^{\prime \prime }}_{i}}\delta {v^{\prime }}_{i}|}_{x_{i}=0}=0,$$

(10) $$\left[{\frac{\partial^{2}h_{V_{2}}}{\partial {v^{\prime \prime }}_{2}\partial x_{2}}|}_{x_{2}=L_{2}}-\int_{0}^{L_{3}}\frac{\partial^{2}h_{T_{3}}}{\partial {\dot{v}}_{2}\left(L_{2}\right)\partial t}dx_{3}\right]\delta v_{2}\left(L_{2}\right)=0,{\frac{\partial^{2}h_{V_{3}}}{\partial {v^{\prime \prime }}_{3}\partial x_{3}}\delta v_{3}|}_{x_{3}=L_{3}}=0,$$

(11) $$\left[{\frac{\partial h_{V_{2}}}{\partial {v^{\prime \prime }}_{2}}\delta {v^{\prime }}_{2}|}_{x_{2}=L_{2}}+\int_{0}^{L_{3}}\frac{\partial^{2}h_{T_{3}}}{\partial {{\dot{v}}^{\prime }}_{2}\left(L_{2}\right)\partial t}dx_{3}\right]\delta {v^{\prime }}_{2}\left(L_{2}\right)=0,{\frac{\partial h_{V_{3}}}{\partial {v^{\prime \prime }}_{3}}\delta {v^{\prime }}_{3}|}_{x_{3}=L_{3}}=0,\;i\in \left\{1,2,3\right\},$$

The axial displacement in the dynamic control in Equation (5) and its boundary condition expressed in Equations (6)–(11) are ignored considering the removal of undamped free vibration and the external excitation terms. The linear undamped homogeneous dynamic control equation is derived from Equation (5) as follows:

(12) $${\ddot{v}}_{1}+\frac{E_{1}J_{1}}{\rho_{1}A_{1}}v_{1}^{\left(4\right)}=0,{\ddot{v}}_{2}+\frac{E_{2}J_{2}}{\rho_{2}A_{2}}v_{2}^{\left(4\right)}+\cos\left(\alpha \right){\ddot{v}}_{1}\left(L_{1}\right)+x_{2}{{\ddot{v}}^{\prime }}_{1}\left(L_{1}\right)=0,$$

(13) $${\ddot{v}}_{3}+\frac{E_{3}J_{3}}{\rho_{3}A_{3}}v_{3}^{\left(4\right)}+\cos\left(\alpha \right)\left[L_{2}{{\ddot{v}}^{\prime }}_{1}\left(L_{1}\right)+{\ddot{v}}_{2}\left(L_{2}\right)\right]+x_{3}\left[{{\ddot{v}}^{\prime }}_{1}\left(L_{1}\right)+{{\ddot{v}}^{\prime }}_{2}\left(L_{2}\right)\right]+{\ddot{v}}_{1}\left(L_{1}\right)=0,$$

where the numbers with small brackets represent a partial derivative to spatial coordinates, the apostrophe represents a partial derivative to the spatial coordinates of the function, and the double dot represents the derivative to the time.

Twelve linear boundary conditions of the three unknown functions in Equations (12) and (13) are expressed as follows:

(14) $${\frac{\partial h_{V_{1}}}{\partial {v^{\prime \prime }}_{1}}\delta {v^{\prime }}_{1}|}_{x_{1}=0}=0,\left[{\frac{\partial h_{V_{1}}}{\partial {v^{\prime \prime }}_{1}}|}_{x_{1}=L_{1}}+\int_{\;0}^{\;L_{2}}\frac{\partial^{2}h_{T_{2}}}{\partial {{\dot{v}}^{\prime }}_{1}\left(L_{1}\right)\partial t}dx_{2}+\int_{\;0}^{\;L_{3}}\frac{\partial^{2}h_{T_{3}}}{\partial {{\dot{v}}^{\prime }}_{1}\left(L_{1}\right)\partial t}dx_{3}\right]\delta {v^{\prime }}_{1}\left(L_{1}\right)=0,$$

(15) $${\frac{\partial^{2}h_{V_{1}}}{\partial {v^{\prime \prime }}_{1}\partial x_{1}}\delta v_{1}|}_{x_{1}=0}=0,\left[{\frac{\partial^{2}h_{V_{1}}}{\partial {v^{\prime \prime }}_{1}\partial x_{1}}|}_{x_{1}=L_{1}}-\int_{\;0}^{\;L_{2}}\frac{\partial^{2}h_{T_{2}}}{\partial {\dot{v}}_{1}\left(L_{1}\right)\partial t}dx_{2}-\int_{\;0}^{\;L_{3}}\frac{\partial^{2}h_{T_{3}}}{\partial {\dot{v}}_{1}\left(L_{1}\right)\partial t}dx_{3}\right]\delta v_{1}\left(L_{1}\right)=0,$$

(16) $${\frac{\partial h_{V_{2}}}{\partial {v^{\prime \prime }}_{2}}\delta {v^{\prime }}_{2}|}_{x_{2}=0}=0,\left[{\frac{\partial h_{V_{2}}}{\partial {v^{\prime \prime }}_{2}}|}_{x_{2}=L_{2}}+\int_{\;0}^{\;L_{3}}\frac{\partial^{2}h_{T_{3}}}{\partial {{\dot{v}}^{\prime }}_{2}\left(L_{2}\right)\partial t}dx_{3}\right]\delta {v^{\prime }}_{2}\left(L_{2}\right)=0,$$

(17) $${\frac{\partial^{2}h_{V_{2}}}{\partial {v^{\prime \prime }}_{2}\partial x_{2}}\delta v_{2}|}_{x_{2}=0}=0,\left[{\frac{\partial^{2}h_{V_{2}}}{\partial {v^{\prime \prime }}_{2}\partial x_{2}}|}_{x_{2}=L_{2}}-\int_{\;0}^{\;L_{3}}\frac{\partial^{2}h_{T_{3}}}{\partial {\dot{v}}_{2}\left(L_{2}\right)\partial t}dx_{3}\right]\delta v_{2}\left(L_{2}\right)=0,$$

(18) $${\frac{\partial h_{V_{3}}}{\partial {v^{\prime \prime }}_{3}}\delta {v^{\prime }}_{3}|}_{x_{3}=0}=0,{\frac{\partial^{2}h_{V_{3}}}{\partial {v^{\prime \prime }}_{3}\partial x_{3}}\delta v_{3}|}_{x_{3}=0}=0,{\frac{\partial h_{V_{3}}}{\partial {v^{\prime \prime }}_{3}}\delta {v^{\prime }}_{3}|}_{x_{3}=L_{3}}=0,{\frac{\partial^{2}h_{V_{3}}}{\partial {v^{\prime \prime }}_{3}\partial x_{3}}\delta v_{3}|}_{x_{3}=L_{3}}=0,$$

The following transformation is introduced:

(19) $$v_{2}^{*}=v_{2}+\cos\left(\alpha \right)v_{1}\left(L_{1}\right)+x_{2}{v^{\prime }}_{1}(L_{1}),$$

(20) $$v_{3}^{*}=v_{3}+v_{1}\left(L_{1}\right)+\cos\left(\alpha \right)\left[L_{2}{v^{\prime }}_{1}\left(L_{1}\right)+v_{2}\left(L_{2}\right)\right]+x_{3}\left[{v^{\prime }}_{1}\left(L_{1}\right)+{v^{\prime }}_{2}\left(L_{2}\right)\right],$$

Equations (12) and (13) are substituted into Equation (5) to obtain the following:

(21) $${\ddot{v}}_{1}^{*}+\frac{E_{1}J_{1}}{\rho_{1}A_{1}}v_{1}^{*}{}^{\left(4\right)}=0,\;{\ddot{v}}_{2}^{*}+\frac{E_{2}J_{2}}{\rho_{2}A_{2}}v_{2}^{*}{}^{\left(4\right)}=0,\;{\ddot{v}}_{3}^{*}+\frac{E_{3}J_{3}}{\rho_{3}A_{3}}v_{3}^{*}{}^{\left(4\right)}=0,$$

The solution to Equation (21) can be expressed as follows:

(22) $$\varphi_{i}^{*}\left(x_{i}\right)=C_{i1}\cos\left(\lambda_{i}x_{i}\right)+C_{i2}\sin\left(\lambda_{i}x_{i}\right)+C_{i3}ch\left(\lambda_{i}x_{i}\right)+C_{i4}sh\left(\lambda_{i}x_{i}\right),\;i\in \left\{1,2,3\right\},$$

Using Equations (14)–(18) and (22), the system frequency characteristic equation can be obtained; then, the frequency eigenvalue can be obtained and the linear frequency can be obtained for each order. By substituting the obtained eigenvalues into Equation (22) and subsequently combining Equations (12) and (13), a modal function can be obtained.

According to the solution of Equation (5) and its corresponding boundary conditions Equations (6)–(11), the two-dimensional (2D) six-degrees-of-freedom beam element is used to divide the resonator. The degrees of freedom of the unit are as follows:

(23) $$\delta_{i}={\left[\begin{array}{cccccc} u_{\varsigma }^{i} & v_{\varsigma }^{i} & \phi_{\varsigma }^{i} & u_{\eta }^{i} & v_{\eta }^{i} & \phi_{\eta }^{i} \end{array}\right]}^{T},$$

where $u_{\varsigma }^{i}$, $v_{\varsigma }^{i}$ and $\phi_{\varsigma }^{i}$ represent the axial degree of freedom, lateral degree of freedom, and section angle of the node ς for unit *i*, respectively. $u_{\eta }^{i}$, $v_{\eta }^{i}$, and $\phi_{\eta }^{i}$ represent the axial degree of freedom, lateral degree of freedom, and section angle of node η for unit *i*, respectively. Superscript T stands for transpose.

The displacement of unit *i* can be written as follows:

(24) $$\left[\begin{array}{c} u_{i}\\ v_{i} \end{array}\right]=N_{i}\delta_{i}=N_{i}{\left[\begin{array}{cccccc} u_{\varsigma }^{i} & v_{\varsigma }^{i} & \phi_{\varsigma }^{i} & u_{\eta }^{i} & v_{\eta }^{i} & \phi_{\eta }^{i} \end{array}\right]}^{T},\;i\in \left\{1,2,3,\ldots ,n\right\},$$

where $N_{i}$ is a 2 × 6 matrix representing the unit functions of the displacement shape, and its expression is as follows:

(25) $$N_{i}=\left[\begin{array}{cccccc} 1-\frac{x}{l_{i}} & 0 & 0 & \left(\frac{x}{l_{i}}\right) & 0 & 0\\ 0 & 1-3{\left(\frac{x}{l_{i}}\right)}^{2}+2{\left(\frac{x}{l_{i}}\right)}^{3} & \left(\frac{x}{l_{i}}\right)-2{\left(\frac{x}{l_{i}}\right)}^{2}+{\left(\frac{x}{l_{i}}\right)}^{3} & 0 & 3{\left(\frac{x}{l_{i}}\right)}^{2}-2{\left(\frac{x}{l_{i}}\right)}^{3} & -{\left(\frac{x}{l_{i}}\right)}^{2}+{\left(\frac{x}{l_{i}}\right)}^{3} \end{array}\right]$$

The kinetic energy and potential energy of unit *i* are as follows:

(26) $$\delta T_{i}=\int \left[\begin{array}{cc} {\dot{u}}_{i} & {\dot{v}}_{i} \end{array}\right]•\left[\begin{array}{c} \delta {\dot{u}}_{i}\\ \delta {\dot{v}}_{i} \end{array}\right]dm_{i}={\dot{\delta }}_{i}^{T}\left(\Pi_{i}^{T}\int N_{i}^{T}\rho_{i}N_{i}dm_{i}\Pi_{i}\right)\delta {\dot{\delta }}_{i},$$

(27) $$\delta V_{i}=\int \varepsilon_{i}\sigma_{i}\delta \varepsilon_{i}dv_{i}=\delta_{i}^{T}\left(\Pi_{i}^{T}\int B_{i}^{T}\sigma_{i}B_{i}dv_{i}\Pi_{i}\right)\delta \delta_{i},$$

where the transformation matrix between the local coordinates of the unit and the global coordinate of the resonator is $\Pi_{i}$, and where the shape function of the axial strain is **B**, the expressions are as follows:

(28) $$\begin{array}{l} \begin{array}{lll} \Pi_{1}=\left[\begin{array}{cccccc} 0 & -1 & 0 & 0 & 0 & 0\\ 1 & 0 & 0 & 0 & 0 & 0\\ 0 & 0 & 1 & 0 & 0 & 0\\ 0 & 0 & 0 & 0 & -1 & 0\\ 0 & 0 & 0 & 1 & 0 & 0\\ 0 & 0 & 0 & 0 & 0 & 1 \end{array}\right], & \Pi_{2}=\left[\begin{array}{cccccc} 1 & 0 & 0 & 0 & 0 & 0\\ 0 & 1 & 0 & 0 & 0 & 0\\ 0 & 0 & 1 & 0 & 0 & 0\\ 0 & 0 & 0 & 1 & 0 & 0\\ 0 & 0 & 0 & 0 & 1 & 0\\ 0 & 0 & 0 & 0 & 0 & 1 \end{array}\right], & \Pi_{3}=\left[\begin{array}{cccccc} 0 & 1 & 0 & 0 & 0 & 0\\ -1 & 0 & 0 & 0 & 0 & 0\\ 0 & 0 & 1 & 0 & 0 & 0\\ 0 & 0 & 0 & 0 & 1 & 0\\ 0 & 0 & 0 & -1 & 0 & 0\\ 0 & 0 & 0 & 0 & 0 & 1 \end{array}\right] \end{array}\\ B_{i}=\left[\begin{array}{cccccc} -\frac{1}{l_{i}} & \bar{y}\left(\frac{6}{l_{i}^{2}}-\frac{12}{l_{i}^{3}}\bar{x}\right) & \bar{y}\left(\frac{4}{l_{i}}-\frac{6}{l_{i}^{2}}\bar{x}\right) & \frac{1}{l_{i}} & \bar{y}\left(\frac{12}{l_{i}^{3}}\bar{x}-\frac{6}{l_{i}^{2}}\right) & \bar{y}\left(\frac{2}{l_{i}}-\frac{6}{l_{i}^{2}}\bar{x}\right) \end{array}\right] \end{array}$$

where $\left(\bar{x},\bar{y}\right)$ represents the local coordinates of unit *i*. To obtain the dynamic control equation of all degrees of freedom of the resonator, unit *i* can be represented by the positioning matrix and the column vector of the total degrees of freedom; the expression is as follows:

(29) $$\delta_{i}=L_{i}P=L_{i}{\left[\begin{array}{ccccccc} u_{0} & v_{0} & \varphi_{0} & \cdots & u_{n} & v_{n} & \varphi_{n} \end{array}\right]}^{T},$$

where **P** is the global generalized coordinate array and $L_{i}$ is the positioning matrix, the expressions are as follows:

(30) $$p={\left[\begin{array}{ccccccc} u_{0} & v_{0} & \varphi_{0} & \cdots & u_{n} & v_{n} & \varphi_{n} \end{array}\right]}^{T},$$

(31) $$\begin{array}{l} \begin{array}{cccc} & & 1 & \begin{array}{cccc} \cdots & & \varsigma & \begin{array}{cccc} \begin{array}{cccc} \begin{array}{cc} \begin{array}{cc} & \cdots \end{array} & \end{array} & & \eta & \end{array} & \cdots & n+1 & \end{array} \end{array} \end{array}\\ L_{i}={\left[\begin{array}{ccccccccccc} 0 & \cdots & 0 & I_{3\times 3} & 0 & \cdots & 0 & 0 & 0 & \cdots & 0\\ 0 & \cdots & 0 & 0 & 0 & \cdots & 0 & I_{3\times 3} & 0 & \cdots & 0 \end{array}\right]}_{6\times 3(n+1),} \end{array}$$

Substituting Equations (23)–(31) into the Hamiltonian equation,

(32) $$\int_{t_{1}}^{t_{2}}\sum_{i=1}^{n}\left(\delta T_{i}-\delta V_{i}\right)dt=0,$$

The dynamic control equation for the global generalized coordinate array is obtained as follows:

(33) $$Μ\ddot{p}+Κp=0,$$

where **M** and **K** are the global mass matrix and global stiffness matrix, the expressions are as follows:

(34) $$M=\sum_{i=1}^{n}L_{i}^{T}\Pi_{i}^{T}\int N_{i}^{T}N_{i}dm_{i}\Pi_{i}L_{i},\;K=\sum_{i=1}^{n}L_{i}^{T}\Pi_{i}^{T}\int B_{i}^{T}\sigma_{i}B_{i}dv_{i}\Pi_{i}L_{i},$$

When the physical parameters of the resonator, such as geometry and material, are specified, the *n*-order natural frequency and vibration mode of the resonator can be calculated by Equation (34).

## 3. Design of the Resonator

**Figure 4:**
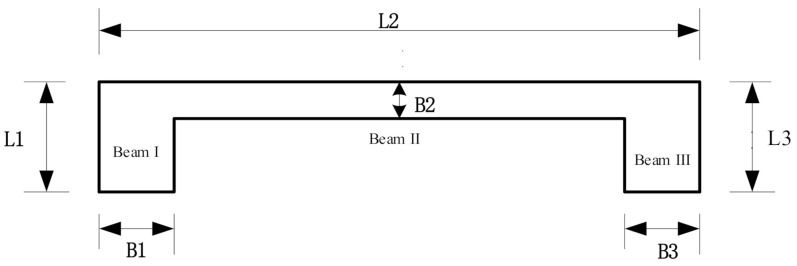
Structural parameters of the resonant beam.

Resonator materials are assumed to be silicon or steel. The relevant parameters for silicon and steel materials are shown in Table 1. The structural parameters for the resonant beam are shown in Figure 4. L1: L2: L3 = 1: PL: 1, B1: B2: B3 = 1: PB: 1, where PL is the length ratio of the three beams, PB is the width ratio of the three beams, and the physical parameters of the three beams are the same. The relationship between frequency and the length, as well as the width, can be obtained by the numerical model Equation (34). The frequency at any point can be compared by using the theoretical model Equations (14)–(18) and (22).

**Figure 5:**
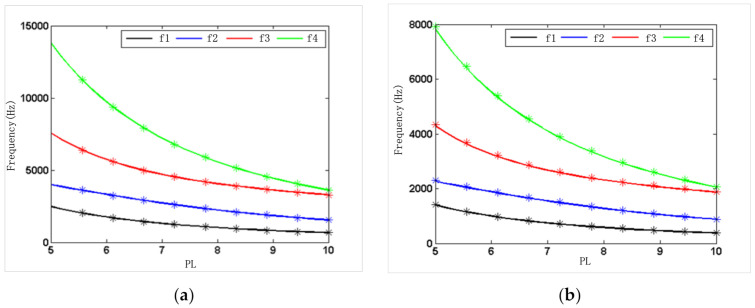
Theoretical and numerical frequencies of the resonator at a fixed-width ratio: (**a**) silicon; (**b**) steel.

**Figure 6:**
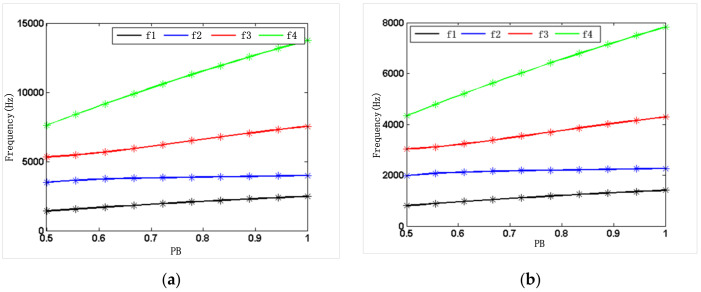
Theoretical and numerical frequencies of the resonator at a fixed-length ratio: (**a**) silicon; (**b**) steel.

As shown in Figure 5 and Figure 6, the solid lines were obtained by the numerical model Equation (34), and the asterisks were derived from theoretical model Equations (14)–(18) and (22). Figure 5 and Figure 6 show the relationship between the resonant frequencies of the first four resonator modes and the fixed-length ratio PL or the width ratio PB under the condition of silicon or steel materials. As shown in Figure 5, when the width ratio of the three beams is fixed in 1, the resonant frequency of each mode and the frequency difference between the modes decrease with the increase of length ratio. As shown in Figure 6, when the length ratio of the three beams is fixed in 6, the natural frequency of each mode and the frequency difference between the modes increase with the increase of width ratio.

Thus, the working mode of the resonator can be quantitatively selected to keep it away from the interference mode. If the second-order mode is selected as the working mode of the resonator, the working mode of the corresponding single beam is the first-order mode. As demonstrated by the results of the simulation and analysis shown in Figure 5 and Figure 6, PL between 5 and 9 was selected, and PB was selected larger than 0.5, which can effectively keep the working mode away from the interference mode.

**Figure 7:**
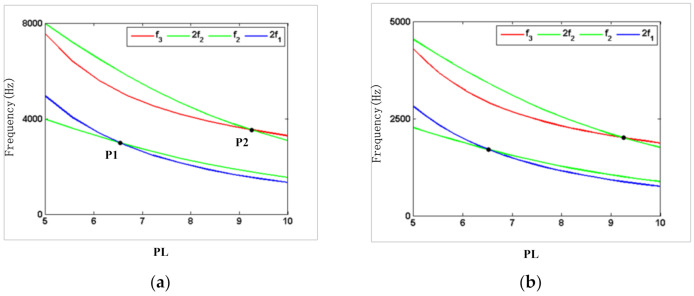
Internal resonant frequency of the resonator at a fixed-width ratio: (**a**) silicon; (**b**) steel.

**Figure 8:**
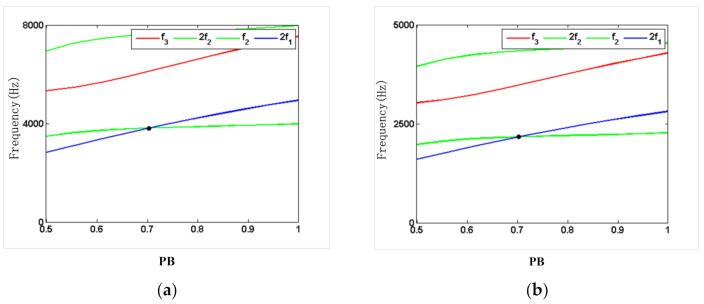
Internal resonant frequency of the resonator at a fixed-length ratio: (**a**) silicon; (**b**) steel.

Depending on the relationship between the higher order mode frequency and the resonator parameters, it was found that there is a multiple relationship between the higher order mode frequency and a certain resonator parameter. When the structural parameter was adjusted to make the second-order mode frequency close to two or three times the first-order mode frequency, the internal resonant conditions between the first two modes were 1:2 or 1:3. Figure 7 and Figure 8 show the internal resonant frequency of the resonator with the silicon and steel material, which were assumed to have a different fixed-length and fixed-width ratios. As shown in Figure 7, under the fixed-width ratio of 1, the second-order mode resonant frequency and the double frequency of the first-order mode resonant frequency intersect at point P1, where the PL is approximately 6.5, indicating that there is a 2:1 internal resonance between the second-order mode and the first-order mode. Under the fixed-width ratio of 1, the third-order mode resonant frequency and the double second-order mode resonant frequency intersect at point P2, where the PL is approximately 9.3, indicating that the resonator parameters have a 2:1 internal resonance between the third-order mode and the second-order mode. Likewise, as can be seen in Figure 8, with the fixed-length ratio of 7, the second-order mode resonant frequency and the double frequency of the first-order mode resonant frequency intersect at the point where the PB is approximately 0.7. It can be seen from the results of the analysis in Figure 7 and Figure 8 that the PL between 6.5 and 9.3 is selected, and the PB smaller than 0.7 is selected, which can avoid the internal resonance points effectively.

Based on the above analysis, physical parameters have little influence on the performance of the accelerometer; hence, the structural parameters of the resonator should be considered essential when optimizing the resonator. According to the mode and frequency analysis, when optimizing resonator structural parameters, the values of PL and PB are determined by theoretical calculation and numerical simulation so that the working mode of the resonator is far away from the interference mode. In addition, the selected PL and PB values of the resonator were verified to avoid the resonance points effectively.

## 4. Optimization of the Resonator

### 4.1. Boundary Conditions

**Figure 9:**
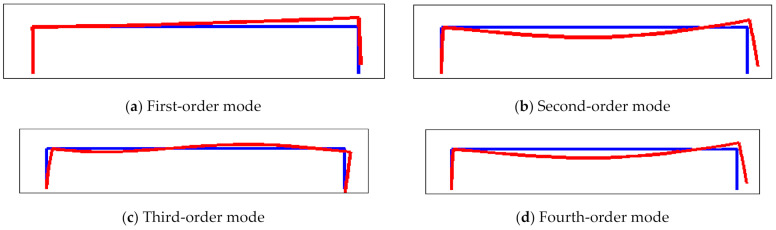
The mode diagram of resonator structure with single-ended fixed support: (**a**) first-order mode; (**b**) second-order mode; (**c**) third-order mode; (**d**) fourth-order mode.

There are two kinds of boundary conditions of a resonator structure, one is single ended and the other is double ended. Figure 9 shows the first-order mode to fourth-order mode of the resonator structure in the case of single-ended fixed support (fixed at the left end, free at the right end). Among them, the second-order mode is the required working mode, but the free end is displaced, so that the excitation energy dissipates to the sensitive structure. When the axial acceleration is perceived, it will form a coupling with the inertia force, which affects the measurement accuracy.

**Figure 10:**
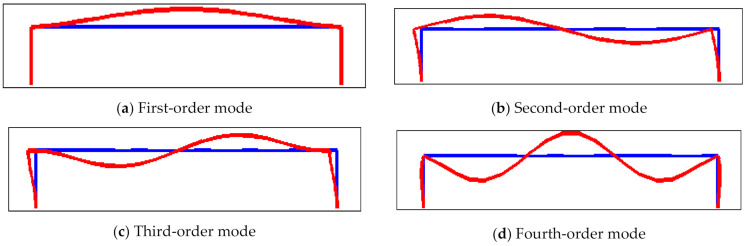
The mode diagram of the resonator structure with double-ended fixed support: (**a**) first-order mode; (**b**) second-order mode; (**c**) third-order mode; (**d**) fourth-order mode.

**Figure 11:**
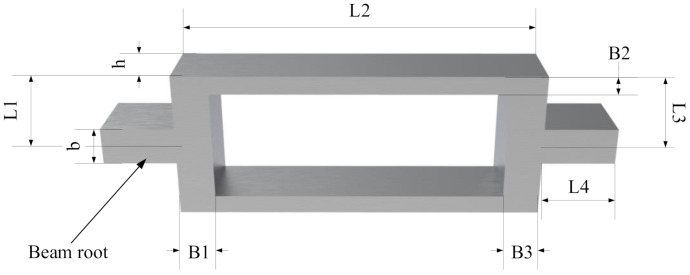
Resonator structure diagram.

Figure 10 shows the first-order mode to fourth-order mode of the resonator structure in the case of double-ended fixed support, and the first-order mode is the working mode. Under the same geometrical parameters, compared with the boundary of single-ended fixed support, the resonant beam with double-ended fixed support is conducive to signal detection. There is no displacement at both ends of the structure, and the energy is concentrated in the middle section, which is in line with the modal shape and design goal required by the ideal resonator. The structural diagram of the resonator is shown in Figure 11. L1, L2, and L3 represent the length of beam I, beam II, and beam III; B1, B2, and B3 represent the width of beam I, beam II, and beam III; h represents the thickness of the resonator; L4 and b represent the length and width of the beam root, respectively.

### 4.2. Structural Parameters

Based on the above mode and frequency analysis, the PL between 5 and 9 was selected, and the PB larger than 0.5 was selected, which can keep the working mode away from the interference mode effectively. The PL between 6.5 and 9.3 was selected, and PB smaller than 0.7 was selected, which can avoid an internal resonance point effectively. Therefore, the length ratio PL was determined to be 8 and the width ratio was determined to be 0.6.

Table 2 shows the resonant beam and beam root parameters and the corresponding stiffness of the three sets of resonators.

**Figure 12:**
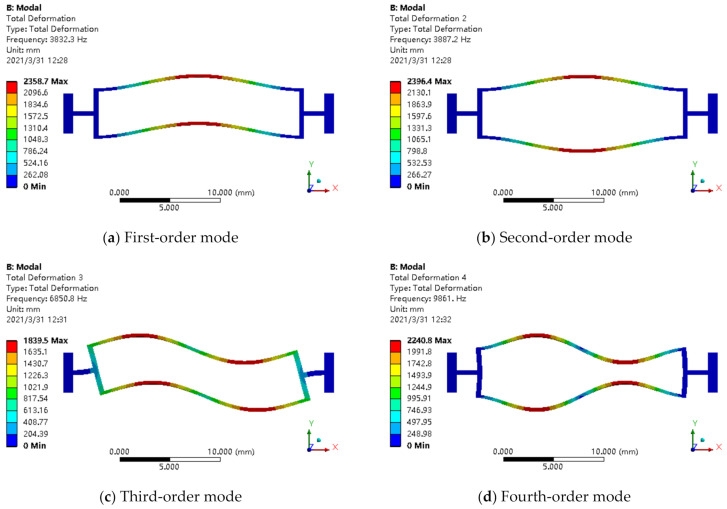
Beam root 1: (**a**) first-order mode; (**b**) second-order mode; (**c**) third-order mode; (**d**) fourth-order mode.

**Figure 13:**
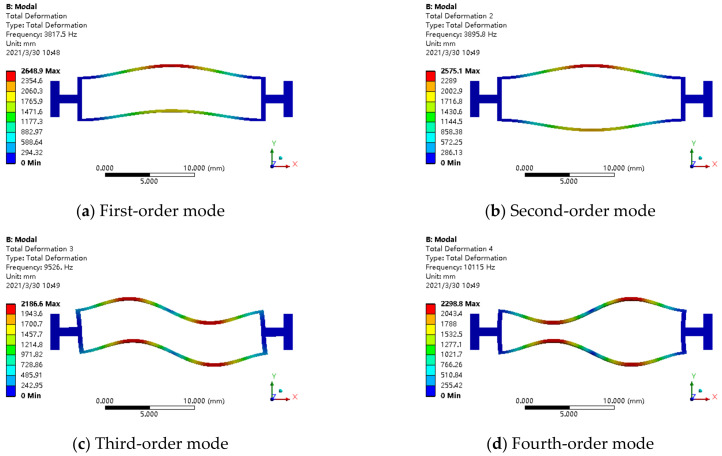
Beam root 2: (**a**) first-order mode; (**b**) second-order mode; (**c**) third-order mode; (**d**) fourth-order mode.

**Figure 14:**
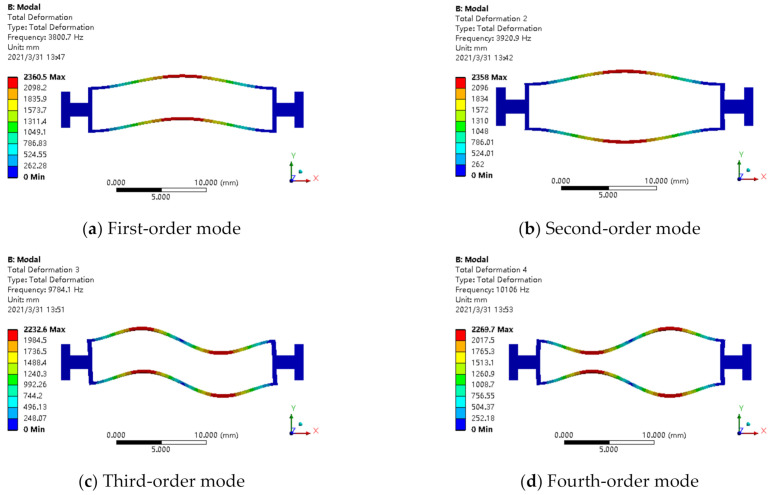
Beam root 3: (**a**) first-order mode; (**b**) second-order mode; (**c**) third-order mode; (**d**) fourth-order mode.

**Figure 15:**
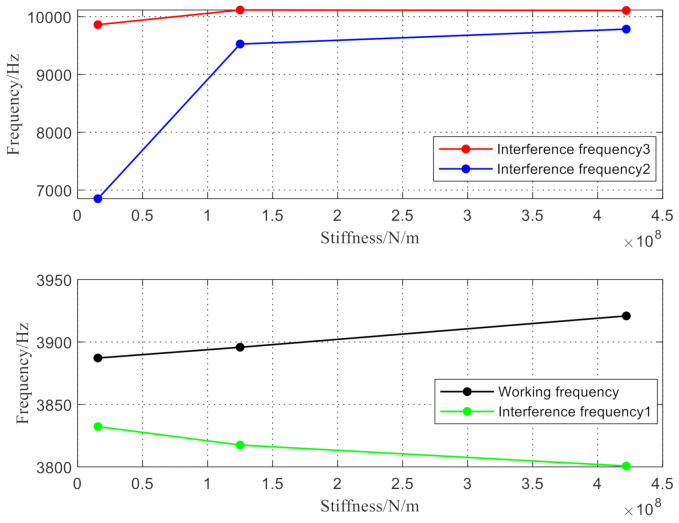
Stiffness frequency of three resonators.

According to the above three groups of structural parameters, the first four modes of each structure were obtained by simulation as shown in Figure 12, Figure 13 and Figure 14. The relationship between the degree of working frequency away from the interference frequency and the resonator root stiffness under different resonator root parameters are shown in Figure 15. The working mode frequency of the resonator was 3901.4 Hz according to $f=\frac{3.75}{L^{2}}\sqrt{\frac{EI}{\rho bh}}$. As shown in Figure 15, the beam root parameters of the resonator were selected from beam root 3, in which the working mode frequencies of the resonator are far away from the interference mode frequencies distinctly.

**Figure 16:**
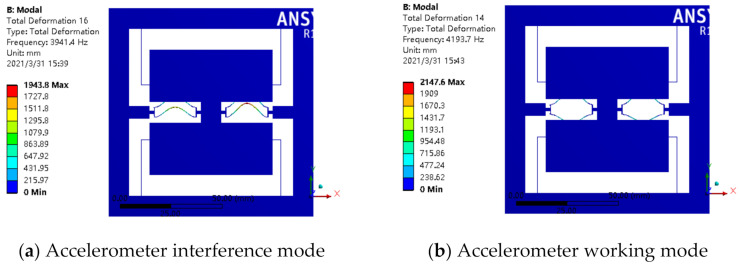
Mode analysis of accelerometer: (**a**) interference mode; (**b**) working mode.

The whole structure of the resonant accelerometer was simulated according to the designed parameters, and the results are as shown in Figure 16, which indicates that the working mode of the accelerometer was far away from the interference mode. The simulated results reflect the effectiveness of the designed resonator parameters.

## 5. Experiments and Discussion

The designed parameters of the resonant accelerometer are shown in Table 3. The resonant accelerometer sample was fabricated from steel materials. In the actual fabrication process, in order to reduce the impact of the fabrication technology precision on the performance of the accelerometer, the micromachining method was adapted to achieve the precise control of the thickness, side wall perpendicularity, smoothness and other process parameters, to ensure the structural dimension accuracy of the accelerometer and improve the sensitivity of the accelerometer. The vibration characteristics of the resonant accelerometer was tested in a series of 1 g acceleration tumbling experiments.

During the accelerometer tumbling experiments, the input acceleration changed according to the sine rule. The corresponding output should also change according to the sine rule. Due to the multiple disturbances, the output was a periodic function and did not change entirely according to the sine rule. The actual output periodic function was broken down by the Fourier series, and the coefficients of the Fourier series could be converted into the coefficients of the output model.

The output model equation can be expressed in the following form:

(35) $$U=K_{0}+K_{1}A_{i}+K_{2}A_{i}^{2}+K_{3}A_{i}^{3},$$

where U is the output of the accelerometer, $K_{0}$ is the partial value, $K_{1}$ is the sensitivity, $K_{2}$ is the nonlinear coefficient of the second order, and $K_{3}$ is the nonlinear coefficient of the third order.

**Figure 17:**
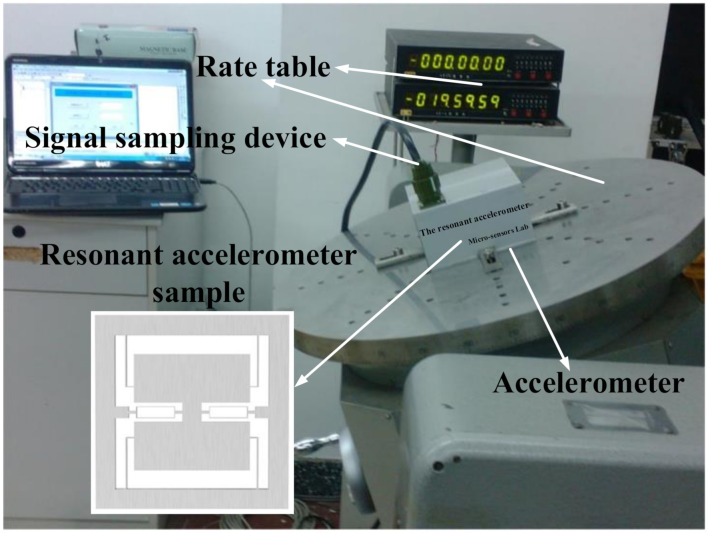
Schematic of the accelerometer 1 g tumbling experiment.

The experimental device shown in Figure 17 is composed of an accelerometer, rate table, and signal sampling device. Initially, vibration isolation and anti-tilt equipment were used to improve the experimental accuracy. The accelerometer was fixed on the rate table, and the observed resonant frequency of the resonator was measured using the signal processing device under the designed rotation angle of the rate table. When there were no acceleration and rotation angles applied to the device, two resonators were driven in to vibrate in their natural resonant frequency. Subsequently, an external rotation angle was applied to the device resulting in a frequency modulated oscillator output that was collected and processed by a signal processing device. Theoretically, the linear output model of the accelerometer can be achieved by measuring the modulated frequency output when the accelerometer is at 1 g. In fact, due to the need for accurate measurement, some special rotation angles were selected for multi-point measurement. In the experiments, the accelerometer device was tested on a rate table providing 36 rotation angles between 0° and 360°. The rate table started from the mechanical zero of the accelerometers and then it was turned clockwise and one way precisely to 0°, 10°, 20°, 30°, 40°, 45°, 50° …, 120°, 130°, 135°, 140° …, 220°, 225°, 230°, …, 310°, 315°, 320°, …, 360°. For each angle within 20 s, 5 output frequencies were selected, then the average of the 5 output frequencies were taken as the output. Finally, the rate table was turned counterclockwise and one way precisely to 360°, 350°, 340°, …, 20°, 10°, 0°, the average of 5 frequencies was also selected out of each angle within 20 s. To improve the accuracy of the measurement, the time of residence at each angle position was equal. The above experiments were repeated four times, and the average of the four measurements was obtained as the output.

The output model of the accelerometer can be obtained by processing the test data as follows:

(36) $$U=-1.553+98.229A_{i}+1.889A_{i}^{2}+1.978A_{i}^{3},$$

**Figure 18:**
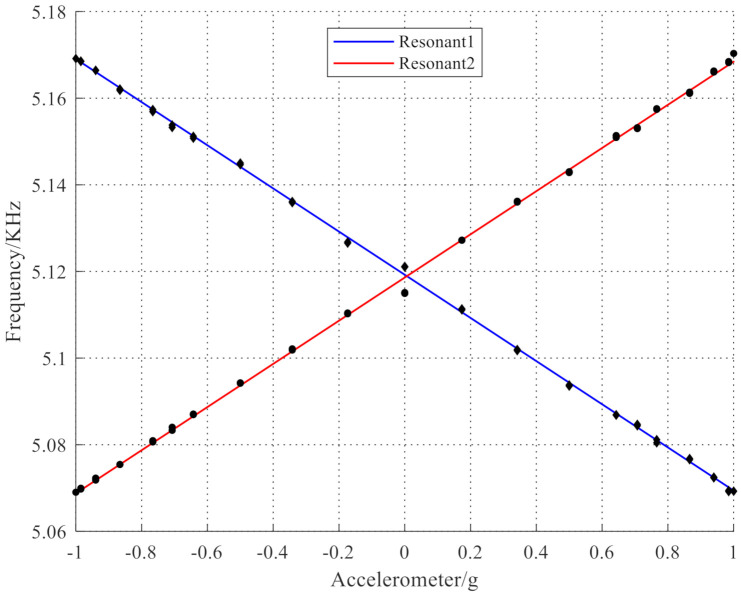
Acceleration frequency of two resonators.

**Figure 19:**
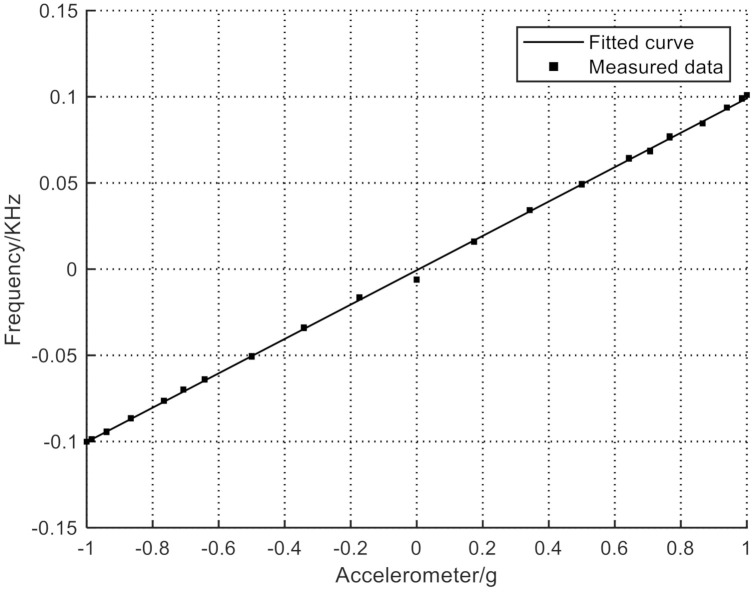
Sensitivity of the resonant accelerometer.

Figure 18 shows that there is a linear relationship between the output frequencies of the tested accelerometer and the acceleration. Figure 19 shows the sensitivity testing curve of the resonant accelerometer sample. Equation (36) shows that the sensitivity of the resonant accelerometer was 98 Hz/g. At the same time, the accelerometer was installed on a two-axis rotating table surface. When the rotating table was leveled, the surface was locked after turning a small angle around the tilt axis. The rotation axis rotated and modulated at a certain angular velocity to subdivide the gravitational acceleration component. After sampling the output of the accelerometer, FFT low-pass filtering was performed. The resolution of the accelerometer was estimated to be 0.917 mg. In addition, the accelerometer was installed under a normal temperature condition; the power was turned on and off repeatedly for multiple sampling measurements after the installation stress was fully released; the bias stability of the accelerometer was estimated to be 1.323 mg/h. The above test results demonstrated that the accelerometer designed according to the proposed mode and frequency analysis method is feasible. In the future work, we will analyze the influence of error sources on the sensitivity of the resonant accelerometer and the solutions to further enhance the sensitivity. We will also consider a new acceleration sensitive mechanism and improve the sensitivity of the accelerometer to achieve an ultra-sensitive accelerometer.

## 6. Conclusions

- According to the working principle of a resonant accelerometer, the resonator was divided into beam I, beam II, and beam III. The shape and displacement of the resonator was described in detail. Using Hamilton’s principle, the undamped dynamic control equation and the corresponding boundary conditions of the resonant beam were obtained. Furthermore, the ordinary differential dynamic equation of the resonant beam with n degrees of freedom was obtained to verify the correctness of the theoretical analysis.
- The structural parameters of the accelerometer were designed and optimized by using resonator mode and frequency analysis; then, they were verified by using a finite element simulation. As the simulation results demonstrated, the working mode of the resonant accelerometer is far away from the interference mode and avoids resonance points effectively.
- From the 1 g acceleration tumbling experiments results of the resonant accelerometer sample, the sensitivity is 98 Hz/g, the resolution is 0.917 mg, and the bias stability is 1.323 mg/h. This shows that the proposed design and optimization method of the resonant accelerometer based on resonator mode and frequency analysis is effective and feasible.
- This research provides an important and novel method for the design and optimization of a resonant accelerometer, which may inspire the use of resonant accelerometers for a variety of motion sensing applications ranging from inertial navigation to vibration monitoring. We will consider a new acceleration sensitive mechanism to achieve an ultra-sensitive accelerometer in the future.

## Figures and Tables

**Table 1:** Physical parameters of the resonator.

	Density/(Kg/m^3^)	Young’s Modulus/(GPa)	Poisson Ratio
Silicon	2300	170	0.278
Steel	7800	211	0.3

**Table 2:** Parameters of the resonant beam and beam root.

Parameters	Resonant Beam	Beam Root 1	Beam Root 2	Beam Root 3
Length (mm)	20	2	2	2
Width (mm)	0.3	0.5	1	1.5
Thickness (mm) Stiffness (*N*/m)	5 3375	5 1.56 × 10^7^	5 1.25 × 10^8^	5 4.22 × 10^8^

**Table 3:** Parameters of the resonant accelerometer.

Parameters	Value	Unit
Density	7.8 × 10^3^	kg/m^3^
Young’s modulus	2 × 10^11^	Pa
External frame (length × width × thickness)	100 × 100 × 5	mm^3^
Poisson ratio	0.3	/
Inner frame (length × width × thickness)	80 × 80 × 5	mm^3^
Proof mass (length × width × thickness)	60 × 50 × 5	mm^3^
Resonant beam (length × width × thickness)	20 × 0.3 × 5	mm^3^
Beam root (length × width × thickness)	2 × 1.5 × 5	mm^3^
Axial force	1.2	*N*
